# Differences in the prognoses of patients referred to an advanced heart failure center from hospitals with different bed volumes

**DOI:** 10.1038/s41598-020-78162-z

**Published:** 2020-12-03

**Authors:** Koichi Narita, Eisuke Amiya, Masaru Hatano, Junichi Ishida, Hisataka Maki, Shun Minatsuki, Masaki Tsuji, Akihito Saito, Chie Bujo, Satoshi Ishii, Nobutaka Kakuda, Mai Shimbo, Yumiko Hosoya, Miyoko Endo, Yukie Kagami, Hiroko Imai, Yoshifumi Itoda, Masahiko Ando, Shogo Shimada, Osamu Kinoshita, Minoru Ono, Issei Komuro

**Affiliations:** 1 Department of Cardiovascular Medicine, Graduate School of Medicine, The University of Tokyo, Hongo 7-3-1, Bunkyo-ku, Tokyo, 113-8655 Japan; 2 Department of Therapeutic Strategy for Heart Failure, The University of Tokyo, Hongo 7-3-1, Bunkyo-ku, Tokyo, 113-8655 Japan; 3 Department of Cardiac Surgery, Graduate School of Medicine, The University of Tokyo, Hongo 7-3-1, Bunkyo-ku, Tokyo, 113-8655 Japan; 4 Department of Organ Transplantation, Graduate School of Medicine, The University of Tokyo, Hongo 7-3-1, Bunkyo-ku, Tokyo, 113-8655 Japan; 5 Department of Cardiovascular Medicine, Saitama Medical Center, Jichi Medical University, 1-847 Amanuma, Omiya-ku, Saitama City, Saitama 330-8503 Japan

**Keywords:** Cardiology, Diseases, Health care

## Abstract

Few reports have discussed appropriate strategies for patient referrals to advanced heart failure (HF) centers with available left ventricular assist devices (LVADs). We examined the association between the characteristics and prognoses of referred patients with advanced HF and the bed volume of the referring hospitals. This retrospective analysis evaluated 186 patients with advanced HF referred to our center for consultation about the indication of LVAD between January 1, 2015, and August 31, 2018. We divided the patients into two groups according to the bed volume of their referring hospital (high bed volume hospitals (HBHs): ≥ 500 beds in the hospital; low bed volume hospitals (LBHs): < 500 beds). We compared the primary outcome measure, a composite of LVAD implantation and all-cause death, between the patients referred from HBHs and patients referred from LBHs. The 186 patients with advanced HF referred to our hospital, who were referred from 130 hospitals (87 from LBHs and 99 from HBHs), had a mean age of 43.0 ± 12.6 years and a median left ventricular ejection fraction of 22% [15–33%]. The median follow-up duration of the patients was 583 days (119–965 days), and the primary outcome occurred during follow-up in 42 patients (43%) in the HBH group and 20 patients (23%) in the LBH group. Patients referred from HBHs tended to require catecholamine infusion on transfer more often than those referred from LBLs (36.5% (HBH), 20.2% (LBL), *P* = 0.021). Kaplan–Meier analysis indicates that the occurrence of the primary outcome was significantly higher in the HBH patients than in the LBH patients (log-rank *P* = 0.0022). Multivariate Cox proportional hazards analysis revealed that catecholamine support on transfer and long disease duration were statistically significant predictors of the primary outcome. Patients from HBHs had a greater risk of the primary outcome. However, the multivariate analysis did not indicate an association between referral from an HBH and the primary outcome. In contrast, catecholamine support on transfer, long duration of disease, and low blood pressure were independent predictors of the primary outcome. Therefore, these should be considered when determining the timing of a referral to an advanced HF center, irrespective of the bed volume of the referring hospital.

## Introduction

In industrialized countries^1^, heart failure (HF) affects 0.4–2.2% of the population and is related to poor quality of life and high mortality rates. HF is also associated with various dysfunctions, leading to enormous financial burdens on the healthcare system^2,3^. In industrialized countries, the incidence of HF is increasing as the average life expectancy increases, and the HF pandemic era is imminent^4^. Furthermore, there are some juvenile cases of medically intractable HF, which is another important issue in medical care for HF^5^. There is an unmet need for the proper allocation of advanced HF therapy in conditions of limited medical resources.

Treatment strategies for advanced HF include a wide range of medical and surgical therapies^6^. These include heart transplantation (HTx) and left ventricular assist device (LVAD) implantation, which have been reported to significantly improve quality of life and survival rates in advanced HF^7^. However, these therapies have limited availability; for example, LVAD implantation is performed only as a bridge to transplantation in Japan, and HTx can be performed only after a long waiting period using LVAD support^8^. In addition, these therapies have not been generalized and can be only performed in limited, specialized facilities. Delays in referrals to specialized facilities for these interventions affect the prognosis and quality of life of patients, as well as the economies of the countries in which they live^9^.

In regard to the indication of LVAD or HTx for advanced HF, the patient should be transferred or referred to specialized facilities that provide these interventions. However, these patient-transfer pathways are wide-ranged and complex. Few reports have investigated the characteristics of patient transfer for considerations of LVAD or HTx.

Therefore, this study aimed to examine the characteristics of patient referrals to LVAD and HTx centers from clinics, community hospitals, and university hospitals to assess the determining factors for the post-transfer clinical course of advanced HF. It is possible that the criteria of transfer might be different in various hospital sizes. In stratifying the referral hospitals in this study, we focused on the bed volume of the hospital. Indeed, there are several reports suggesting that hospital bed volume has some degree of impact on the post-transfer clinical course of patients with advanced HF^10,11^. We investigated the different characteristics of referred patients with advanced HF according to the bed volume of the referring hospital.

## Methods

### Study protocol

The University of Tokyo Hospital is an approved HTx facility and has performed approximately 15–20 HTx procedures annually. This facility also performs LVAD surgery, making it the most active center for these interventions within the Kanto area, which is located in the north central region of the main island of Japan and is approximately 30,000 km^2^. In this study, we recruited consecutive patients who were referred to our hospital for advanced HF with consideration of the indication for an advanced intervention, including LVAD and HTx, between January 1, 2015, and August 31, 2018.

The exclusion criteria are as follows: age greater than 65 years (because there is no indication for transplantation in these patients), age less than 18 years, hospital transfer for an already-planned LVAD operation, and diagnosis of diseases other than HF that could cause volume overload, such as end-stage renal disease requiring hemodialysis and severe liver disease. We excluded patients with ischemic heart disease and patients with severe valve disease, if they could be improved by the treatment for each complication, such as coronary or valve interventions. We also excluded patients who were consulted from only documents due to a lack of information. The diagnosis of HF was confirmed if HF was diagnosed as the primary admitting diagnosis by the documented physical examination and laboratory and radiologic findings. The study protocol conformed to the tenets of the Declaration of Helsinki and was reviewed and approved by the institutional review board at the University of Tokyo (approval number: 2650). Informed consent was obtained from all patients, in accordance with the protocol approved by ethics committee.

### Follow-up

In this study, follow-up examinations were completed on December 31, 2019. The patients’ survival was followed up from the date of patient transfer until death or HTx. We also evaluated the date of implantable LVAD operation, survival with LVAD implantation, and survival without LVAD implantation. The median follow-up duration was 583 (119–965) days. The primary outcome was a composite of implantable LVAD implantation and all-cause death. We also examined all-cause death inclusive of deaths after LVAD implantation as a secondary outcome. None of the patients included in this study underwent HTx without implantable LVAD implantation. The implantation of extracorporeal LVAD was not counted as an event, but we evaluated it as a bridging intervention toward implantable LVAD. In addition, the indication of LVAD was determined by an advanced HF therapeutics team including cardiologists, a surgeon, nurses, and a transplant coordinator. Timing of the LVAD implantation was determined by whether the patient developed a progressive decline in end-organ function despite receiving the maximal treatment.

### Data collection

Patient characteristics, including medications, were collected at the time of transfer. The beta-blocker dose at baseline was standardized into bisoprolol equivalents^12^, and the loop diuretic dose was standardized into furosemide equivalents^13^, both of which were analyzed as continuous variables. The duration of the disease was defined as the time from the date of the first onset of HF symptoms according to the medical charts to the time of transfer. For the laboratory data, fasting blood samples were collected at the time of patient transfer, and the laboratory data were assessed using standard laboratory methods at the University of Tokyo Hospital. We examined the most recent transthoracic echocardiography data available around the time of transfer. “Direct transfer” means that hospitalized patients in other hospitals were transferred and admitted into our hospital directly. We also examined the status of the patient during direct transfer, and this assessment included statuses of “with catecholamine infusion,” “with intra-aortic balloon pumping (IABP),” and “with extracorporeal membrane oxygenation (ECMO).” The data generated and analyzed in the current study are not publicly available but are available from the corresponding author on reasonable request.

### Statistical analysis

Data are presented as the mean ± standard deviation or as the median (interquartile range). The statistical analysis was performed using JMP version Pro 14. Student’s t-test or the Mann–Whitney U test was used to compare continuous variables, and Fisher’s exact test was used to compare categorical variables. Log-rank tests using the Kaplan–Meier estimator were performed to determine the survival rate of the patients, in which time zero was set as the time of transfer. The level of significance was set to 5%, and all reported *P* values and confidence intervals (CIs) were calculated as two-sided. The cutoff value of each variable for the hazard analysis was calculated using a receiver operating characteristic curve. We chose values that maximized the sum of sensitivity and specificity as the cutoff values to calculate the area under the curve. According to the analysis of risk factors for the primary outcome, a multivariate Cox proportional hazards analysis including the factor of hospital bed volume was performed using a backward selection procedure. Of note, a forward selection procedure resulted in the same selection of independent parameters. According to the analysis of risk factors for the secondary outcome, multivariate Cox proportional hazards analysis was performed using a backward selection procedure. A forward selection procedure resulted in the same results.

## Results

### Baseline characteristics

**Figure 1:**
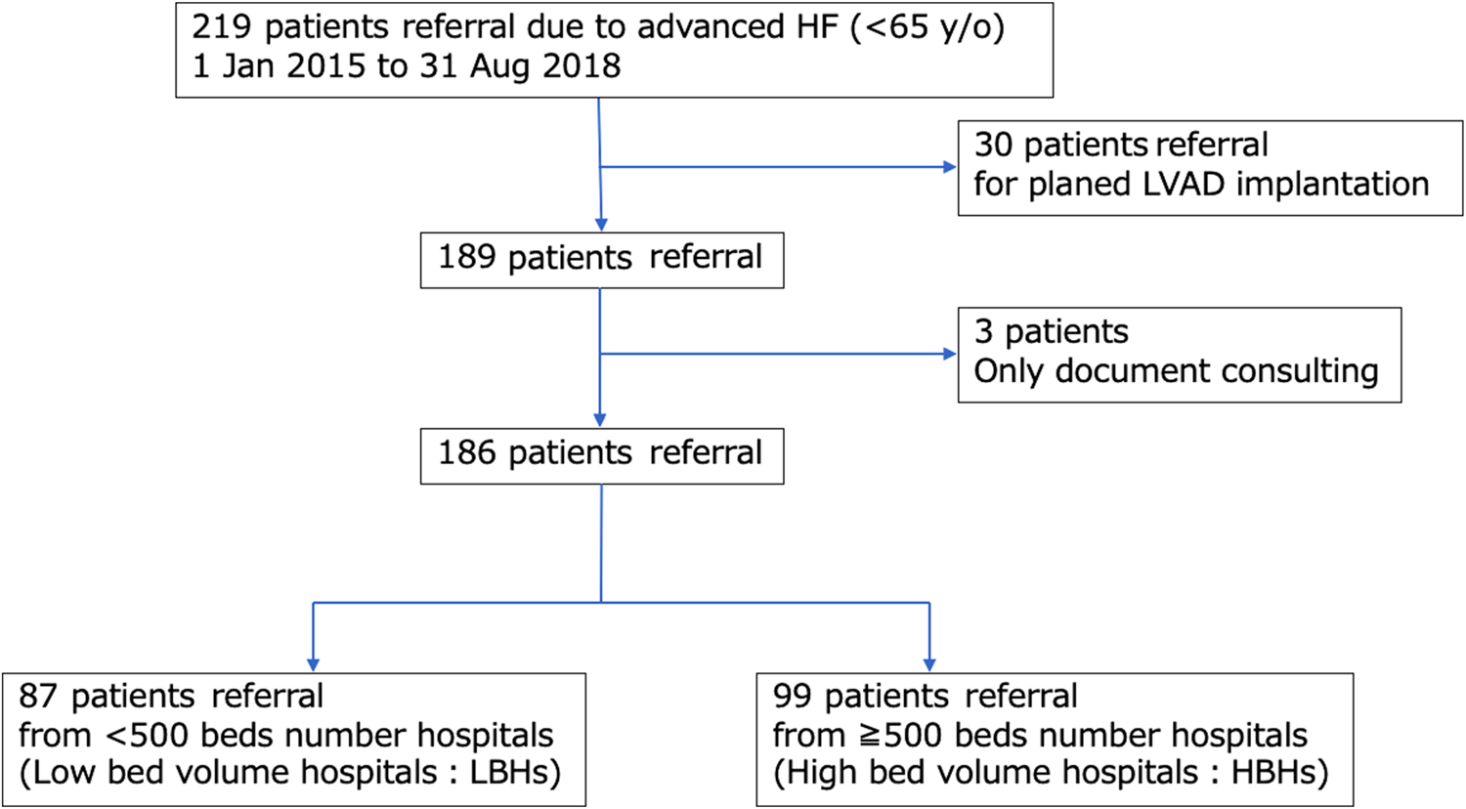
Flowchart of the study depicting the referral of advanced HF patients from other hospitals to our advanced HF center. Certain exclusion criteria were applied. HF, heart failure; LVAD, left ventricular assist device.

**Figure 2:**
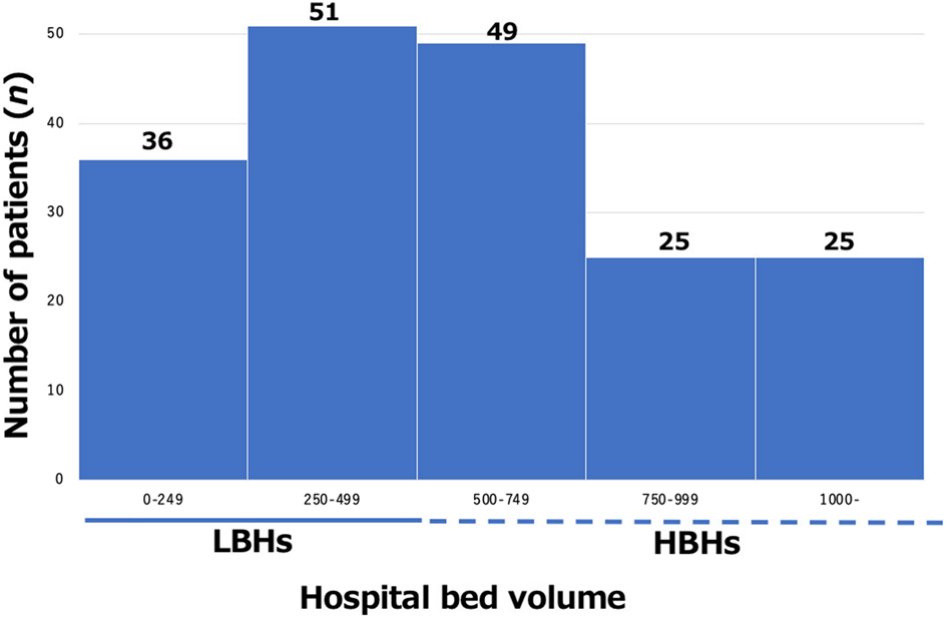
Numbers of HF patients referred to our advanced HF center from HBHs and LBHs. HF, heart failure; LBHs, low bed volume hospitals; HBHs, high bed volume hospitals.

A total of 186 patients with advanced HF were assessed in this analysis after 33 patients were excluded according to the excluding criteria (Fig. 1). Figure 2 shows the number of hospital beds for the hospitals from which the advanced HF patients were transferred to our center. The median number of hospital beds was 504. The mean age of the included advanced HF patients was 43.0 ± 12.6 years; 72.0% of the patients were male; 67.2% of the patients had dilated cardiomyopathy; the median (25th percentile to 75th percentile) left ventricular ejection fraction (LVEF) of the patients was 22% (15–33%); and 24.6% of the patients were classified as New York Heart Association (NYHA) functional class II, whereas 75.4% were NYHA functional class III or IV. Beta-blockers were taken by 73.4% of the patients, angiotensin-modulating agents by 62.0%, and mineralocorticoid receptor antagonists by 61.4%. Furthermore, 22.2% had an implantable cardioverter defibrillator/cardiac resynchronization therapy defibrillator (ICD/CRTD). A total of 38% of the patients were directly transferred to our hospital, 73.2% of whom received a continuous infusion of catecholamine; additionally, 28.1% received IABP, and 18.3% received ECMO. Regarding the implantation of an extracorporeal LVAD, 11 patients had extracorporeal LVAD as a bridging therapy toward the explanation of it (N = 3) or implantable LVAD implantation (N = 4).

### Differences in patient characteristics between the HBH and LBH groups

Next, we divided the patients into two groups based on the bed volume of the hospital from which they were referred: the high bed volume hospital (HBH, ≥ 500 beds) group and low bed volume hospital (LBH, < 500 beds) group. We compared the baseline characteristics between the patients referred from LBHs and the patients referred from HBHs (Table 1).

**Table 1:** Baseline characteristics of HF patients with respect to the bed volume of the referring hospital (more than or equal to 500 beds (HBHs) or less than 500 beds (LBHs)).

	Low bed volume (LBHs)	High bed volume (HBHs)	*P*-value
	(N = 87: 47%)	(N = 99: 53%)	
Age (years)	45.0 [36.0–53.0]	44.0 [35.0–52.0]	0.60
Sex (male)	64 (73.6%)	70 (70.7%)	0.74
BMI (kg/m^2^)	22.4 [20.0–26.3]	21.8 [19.3–24.6]	0.13
BSA (DuBois, m^2^)	1.75 [1.58–1.88]	1.69 [1.59–1.83]	0.33
Systolic blood pressure (mmHg)	104 [92–117]	99 [86–108]	0.073
Diastolic blood pressure (mmHg)	62 [56–70]	60 [52–68]	0.14
Heart rate (beats/min)	78 [72–89]	80 [68–100]	0.39
**NYHA**			
II, *n* (%)	24 (28.6%)	20 (21.1%)	
III, *n* (%)	34 (40.5%)	31 (32.6%)	
IV, *n* (%)	26 (30.9%)	44 (46.3%)	
**Stage**			
B, *n* (%)	7 (8.3%)	2 (2.1%)	
C, *n* (%)	55 (65.5%)	47 (49.5%)	
D, *n* (%)	26 (30.9%)	44 (46.3%)	
**Medical history**			
Hypertension	19 (21.8%)	17 (17.2%)	0.46
Diabetes	19 (21.8%)	18 (18.2%)	0.58
Dyslipidemia	30 (34.5%)	23 (23.2%)	0.10
Smoking	45 (51.7%)	46 (46.9%)	0.56
Atrial fibrillation	17 (19.5%)	18 (18.4%)	0.85
Stroke	5 (5.8%)	11 (11.1%)	0.29
ICD/CRTD	10 (11.5%)	31 (31.6%)	0.0013*
Duration of HF (days)	350 [61–2635]	1658 [89–4223]	0.041*
**Etiology**			
DCM, *n* (%)	59 (67.8%)	66 (66.7%)	0.88
HCM, *n* (%)	6 (6.9%)	10 (10.2%)	0.45
RCM, *n* (%)	0 (0.0%)	2 (2.0%)	0.50
ICM, *n* (%)	10 (11.5%)	6 (6.1%)	0.20
Myocarditis	6 (6.9%)	12 (12.2%)	0.32
Others^a^	6 (6.9%)	4 (4.0%)	0.52
Family history	12 (13.8%)	11 (11.1%)	0.66
**Direct transfer**	23 (26.4%)	48 (48.5%)	0.0025*
With catecholamine support	17 (20.2%)	35 (36.5%)	0.021*
With IABP support	3 (3.6%)	17 (17.7%)	0.0034*
With ECMO support	2 (2.4%)	11 (11.3%)	0.022*
LVAD implantation	15 (18.3%)	35 (39.3%)	0.0040*
All-cause death	5 (6.1%)	8 (8.89%)	0.57
Primary outcome^b^	20 (23.0%)	42 (43.3%)	0.0048*
Cardiac rehabilitation	60 (70.6%)	73 (77.7%)	0.31
**Echocardiographic data**			
LVEF (%)	24.0 [16.8–33.0]	20.0 [14.8–33.0]	0.24
LVDd (mm)	65.1 ± 11.6	64.1 ± 14.1	0.63
LVDs (mm)	58.0 ± 13.3	56.5 ± 16.0	0.52
IVST (mm)	8.0 [7.0–9.0]	8.0 [7.0–10.0]	0.81
PWT (mm)	8.0 [7.0–10.0]	8.0 [6.0–10.0]	0.36
LAD (mm)	44.7 ± 9.5	45.1 ± 10.6	0.81
**Medication management on referral**			
Beta-blocker	65 (75.6%)	70 (71.4%)	0.62
Bisoprolol (mg/day)^c^	0.45 ± 1.49	0.72 ± 1.77	0.32
ACEi/ARB	51 (59.3%)	63 (64.3%)	0.53
Statin	24 (28.6%)	14 (14.4%)	0.028*
Diuretics	67 (77.9%)	78 (79.6%)	0.86
Furosemide (mg/day)^d^	27.4 ± 29.3	29.2 ± 31.1	0.73
MRA	50 (58.1%)	63 (64.3%)	0.45
Tolvaptan	21 (24.4%)	34 (34.7%)	0.15
Carperitide	5 (5.8%)	6 (6.1%)	1.00
SGLT2 inhibitor	2 (2.3%)	1 (1.0%)	0.60
**Laboratory data**			
Albumin (g/dL)	4.1 [3.6–4.4]	3.9 [3.4–4.2]	0.037*
Total protein (g/dL)	7.0 [6.5–7.3]	6.8 [6.1–7.1]	0.047*
AST (U/L)	25.0 [20.0–39.5]	26.0 [19.5–42.0]	0.93
ALT (U/L)	24.0 [16.0–45.0]	23.0 [15.5–37.5]	0.66
γGTP (U/L)	56.0 [29.0–124.5]	58.0 [34.5–110.5]	0.99
Total cholesterol (mg/dL)	164.1 ± 43.3	159.4 ± 47.0	0.51
Total bilirubin (mg/dL)	1.00 [0.70–1.60]	1.00 [0.70–1.30]	0.60
Creatinine (mg/dL)	0.93 [0.79–1.19]	0.97 [0.73–1.21]	0.94
eGFR (mL/min/1.73 m^2^)	63.5 [49.1–85.1]	66.8 [51.3–80.3]	0.90
Sodium (mmol/L)	138.4 ± 3.4	137.4 ± 4.1	0.059
Potassium (mmol/L)	4.27 ± 0.48	4.29 ± 0.58	0.81
CRP (mg/dL)	0.14 [0.05–0.55]	0.21 [0.06–1.62]	0.086
White blood cells (× 1000/μL)	6.8 [5.3–8.8]	6.9 [5.6–8.6]	0.67
Lymphoid (× 1000/μL)	1.4 [1.0–1.8]	1.6 [1.2–2.1]	0.11
Hemoglobin (g/dL)	14.2 ± 2.1	13.2 ± 2.2	0.0015*
Platelet (× 10,000/μL)	22.6 ± 6.4	20.5 ± 8.3	0.062
Hemoglobin A1c (%)	6.1 [5.7–6.6]	5.9 [5.5–6.2]	0.016*
BNP (pg/mL)	408.4 [126.3–863.5]	422.2 [161.1–968.3]	0.67

BMI, body mass index; BSA, body surface area; NYHA, New York Heart Association functional classification; ICD/CRTD, implantable cardioverter defibrillator/cardiac resynchronization therapy defibrillator; DCM, dilated cardiomyopathy; HCM, hypertrophic cardiomyopathy; RCM, restrictive cardiomyopathy; ICM, ischemic cardiomyopathy; IABP, intra-aortic balloon pumping; ECMO, extracorporeal membrane oxygenation; LVAD, left ventricular assist device; LVEF, left ventricular ejection fraction; LVDd, left ventricular end-diastolic dimension; LVDs, left ventricular end-systolic dimension; IVST, interventricular septum end-diastolic thickness; PWT, posterior left ventricular wall end-diastolic thickness; LAD, left atrial dimension; ACEi, angiotensin-converting enzyme inhibitor; ARB, angiotensin II receptor blocker; MRA, mineralocorticoid receptor antagonist; SGLT2, sodium glucose transporter 2; AST, aspartate aminotransferase; ALT, alanine aminotransferase; γGTP, γ-glutamyl transpeptidase; eGFR, estimated glomerular filtration rate; CRP, C-reactive protein; BNP, brain natriuretic peptide.

*P < 0.05.

^a^Others include sarcoidosis, structural heart disease, arrhythmogenic right ventricular cardiomyopathy, anthracycline-induced cardiomyopathy, and tachycardia-induced cardiomyopathy.

^b^Primary outcome; a composite of implantable LVAD implantation and all-cause death.

^c^Standardized with bisoprolol equivalents.

^d^Standardized with furosemide equivalents.

This study included patients referred from 70 LBHs (62% of these hospitals have their own coronary care unit) and 60 HBHs (100% of these hospitals have their own coronary care unit). The percentage of university hospitals was significantly different between the two groups (6% (LBHs) vs. 58% (HBHs)). The median age of the patients was 45.0 [36.0–53.0] years (LBHs) versus 44.0 [35.0–52.0] years (HBHs) (*P* = 0.60), and 73.6% (LBHs) vs 70.7% (HBHs) of the patients were male (*P* = 0.74). There were no significant differences in the etiologies of HF and previous medical histories of the patients between the two groups. In contrast, there were significant differences in the albumin levels (4.1 [3.6–4.4] g/dL (LBHs) vs. 3.9 [3.4–4.2] g/dL (HBHs); *P* = 0.037) and hemoglobin levels (14.2 ± 2.1 g/dL (LBHs) vs. 13.2 ± 2.2 g/dL (HBHs); *P* = 0.0015), which are surrogate markers of nutritional state, of the patients between the two groups. The duration of diagnosed HF was longer in the patients from HBHs than in patients from LBHs (350 [61–2635] days (LBHs) vs. 1658 [89–4223] days (HBHs); *P* = 0.041). Furthermore, there was a higher proportion of patients who had an ICD/CRTD from HBHs than those from LBHs (11.5% (LBHs) vs. 31.6% (HBHs); *P* = 0.0013). According to the transfer, catecholamine infusion (20.2% (LBHs) vs. 36.5% (HBHs); *P* = 0.0012), IABP (3.6% (LBHs) vs. 17.7% (HBHs); *P* = 0.0020), and ECMO (2.4% (LBHs) vs. 11.3% (HBHs); *P* = 0.018) were observed more frequently in the patients from HBHs. There were no significant differences in the rate of medication with other than statins for patients between the two groups (28.6% (LBHs) vs. 14.4% (HBHs); *P* = 0.028). Interestingly, no differences in echocardiographic parameters were observed between the LBHs and HBHs.

### Clinical course after patient transfer

During the duration of follow-up in this study, there were 12 deaths among the patients (three after extracorporeal LVAD implantation and nine who did not undergo LVAD implantation) and 50 patients who underwent LVAD implantation. We also detected death in one patient after implantable LVAD implantation.

**Figure 3:**
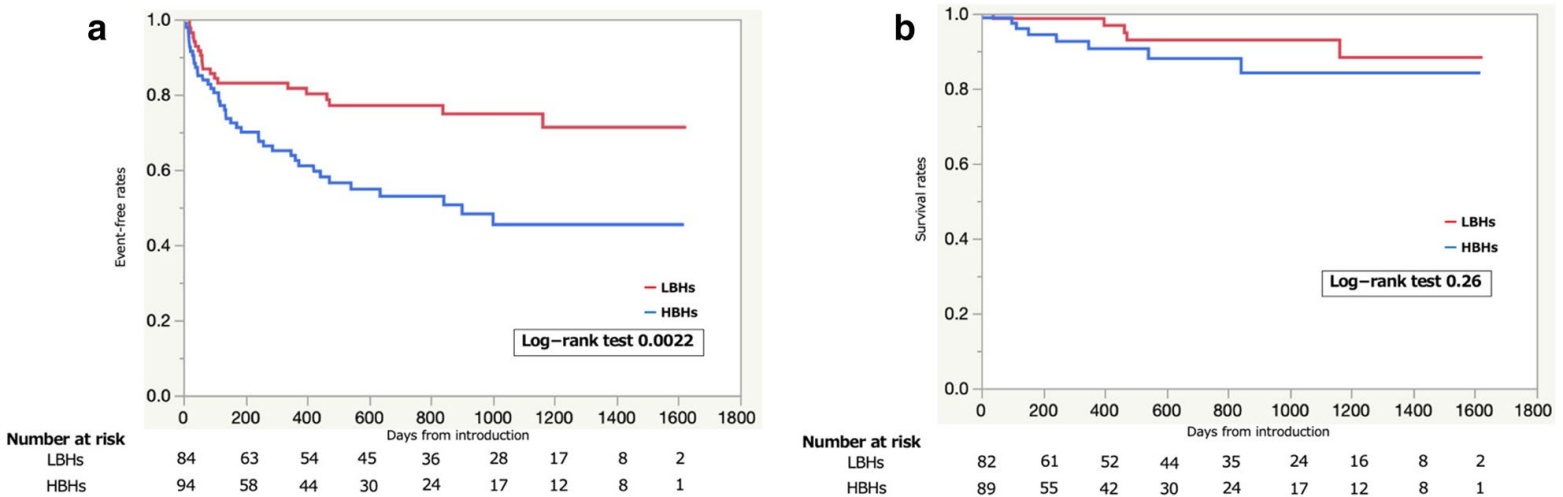
(**a**) Differences in event-free survival curves of the primary outcome measures, including LVAD implantation and death, between patients referred from HBHs and patients referred from LBHs. (**b**) Differences in event-free survival curves of the secondary outcome measures between patients referred from HBHs and patients referred from LBHs. LVAD, left ventricular assist device; LBHs, low bed volume hospitals; HBHs, high bed volume hospitals.

We investigated the impact of hospital bed volume on event occurrence of the primary outcome. The results show that the patients from HBHs had a higher occurrence of the primary outcome during follow-up than did the patients from LBHs (Kaplan–Meier log-rank *P* = 0.0022) (Fig. 3a). However, there was no significant difference in survival rate between these two groups (Fig. 3b).

### Critical factors that affected the primary and secondary outcomes

Next, we performed monovariate and multivariate Cox proportional hazards analyses to determine the factors affecting the primary outcome, focusing on the impact of bed volume of the referring hospital (Table 2).

**Table 2:** Monovariate and multivariate Cox proportional analysis of factors that determined the risk of the primary outcome.

Parameter	Monovariate		Multivariate	
	Hazard ratio	*P*-value	Hazard ratio	*P*-value
	(95% CI)		(95% CI)	
Age (< 44 years)	1.24 (0.75–2.05)	0.39		
Hospital bed number (≥ 500 beds)	2.25 (1.32–3.84)	0.0029*	1.30 (0.72–2.34)	0.38
Duration of HF (≥ 684 days)	4.51 (2.38–8.53)	< 0.0001*	2.41 (1.16–5.00)	0.0019*
Systolic BP (< 100 mmHg)	3.28 (1.80–5.97)	0.0001*	2.36 (1.25–4.49)	0.0085*
Heart rate (≥ 80 bpm)	1.79 (1.07 − 3.00)	0.028*		
With catecholamine support	7.21 (4.29–12.1)	< 0.0001*	5.56 (3.11–9.95)	< 0.0001*
With IABP support	1.64 (0.83–3.22)	0.15		
With ECMO support	1.97 (0.90–4.33)	0.091		
ICD/CRTD	3.76 (2.26–6.24)	< 0.0001*	1.86 (1.04–3.33)	0.038*
LVEF (< 17%)	2.08 (1.24–3.48)	0.0054*		
Albumin (< 4.3 g/dL)	3.07 (1.55–6.05)	0.0013*		
ALT (≥ 68 U/L)	2.58 (1.41–4.70)	0.0020*	1.93 (1.00–3.72)	0.049*
eGFR (< 78.4 mL/min/1.73 m^2^)	1.72 (0.93–3.20)	0.083		
Total cholesterol (< 160 mg/dL)	2.51 (1.47–4.29)	0.0008*		
BNP (≥ 418 pg/mL)	3.52 (2.00–6.18)	< 0.0001*		
Sodium level (< 138 mmol/L)	2.69 (1.61–4.51)	0.0002*		
Hemoglobin (< 14.1 g/dL)	2.60 (1.49–4.57)	0.0008*		
Lymphoid (< 1700/μL)	3.45 (1.78–6.68)	0.0002*		

CI, confidence interval; LVAD, left ventricular assist device; HF, heart failure; BP, blood pressure; IABP, intra-aortic balloon pumping; ECMO, extracorporeal membrane oxygenation; ICD/CRTD, implantable cardioverter defibrillator/cardiac resynchronization therapy defibrillator; LVEF, left ventricular ejection fraction; ALT, alanine aminotransferase; eGFR, estimated glomerular filtration rate; BNP, brain natriuretic peptide.

*P < 0.05.

**Figure 4:**
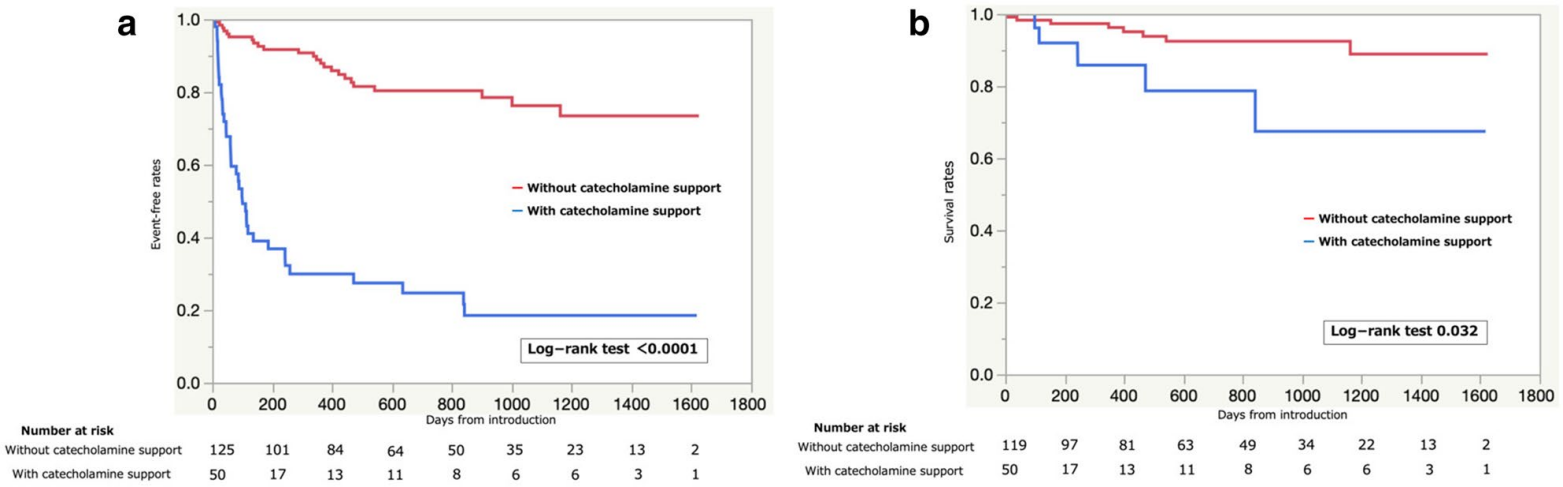
(**a**) Differences in event-free survival curves of the primary outcome measures, including LVAD implantation and death, between patients with and patients without catecholamine infusion during transfer. (**b**) Differences in event-free survival curves of the secondary outcome measures between patients with and patients without catecholamine infusion during transfer. LVAD, left ventricular assist device.

Monovariate analysis indicated that the patients referred from HBHs had a higher risk of the primary event compared to the patients from LBHs. During transfer, catecholamine infusion had the greatest impact on the primary outcome of all other factors, and this was also exemplified in the survival curve analysis (Fig. 4a). However, neither the IABP nor ECMO resulted in a higher risk for the primary outcome. Additionally, systolic blood pressure, LVEF on echocardiography, lymphocyte count, and the levels of albumin, alanine aminotransferase, total cholesterol, brain natriuretic peptide (BNP), sodium, and hemoglobin corresponded to varying risks for the primary event. The multivariate analysis indicated that catecholamine infusion, HF duration, low systolic blood pressure, ICD/CRTD, and alanine aminotransferase elevation corresponded to a significantly higher risk for outcomes occurring in the future. However, the difference in hospital bed volume between the two groups lost statistical significance in predicting the primary outcome after the multivariate analysis was performed. We also analyzed the predictive factors of all-cause death (secondary outcome), which demonstrated that catecholamine infusion during transfer was a statistically significant predictor of secondary outcomes (Table 3). The Kaplan–Meier analysis demonstrated that there was a higher risk of all-cause death in the group with catecholamine infusion during transfer than those without catecholamine infusion during transfer (Fig. 4b).

**Table 3:** Monovariate and multivariate Cox proportional analysis of factors that determined the risk of the secondary outcome.

Parameter	Monovariate		Multivariate	
	Hazard ratio	*P*-value	Hazard ratio	*P*-value
	(95% CI)		(95% CI)	
Age (< 44 years)	0.88 (0.64–1.21)	0.44		
Hospital bed number (≥ 500 beds)	1.89 (0.62–5.81)	0.27		
Duration of HF (≥ 684 days)	1.97 (1.42–2.72)	< 0.0001*	1.96 (1.41–2.73)	< 0.0001*
Systolic BP (< 100 mmHg)	2.10 (0.63–7.00)	0.23		
Heart rate (≥ 80 bpm)	1.74 (0.55–5.49)	0.34		
With catecholamine support	1.84 (1.30–2.63)	0.0007*	1.81 (1.27–2.57)	0.0011*
With IABP support	5.08 (1.66–15.6)	0.0045*		
With ECMO support	10.2 (3.33–31.5)	0.0001*		
ICD/CRTD	2.55 (0.76–8.56)	0.13		
LVEF (< 17%)	2.48 (0.79–7.83))	0.12		
Albumin (< 4.3 g/dL)	3.17 (0.69–14.5)	0.14		
ALT (≥ 68 U/L)	4.94 (1.48–16.5)	0.0094*		
eGFR (< 78.4 mL/min/1.73 m^2^)	1.59 (0.43–5.90)	0.49		
Total cholesterol (< 160 mg/dL)	2.71 (0.79–9.34)	0.11		
BNP (≥ 418 pg/mL)	1.45 (1.05–1.99)	0.023*		
Sodium level (< 138 mmol/L)	1.44 (0.46–4.56)	0.53		
Hemoglobin (< 14.1 g/dL)	11.7 (1.51–91.0)	0.019*		
Lymphoid (< 1700/μL)	2.82 (0.76–10.4)	0.12		

CI, confidence interval; LVAD, left ventricular assist device; HF, heart failure; BP, blood pressure; IABP, intra-aortic balloon pumping; ECMO, extracorporeal membrane oxygenation; ICD/CRTD, implantable cardioverter defibrillator/cardiac resynchronization therapy defibrillator; LVEF, left ventricular ejection fraction; ALT, alanine aminotransferase; eGFR, estimated glomerular filtration rate; BNP, brain natriuretic peptide.

*P < 0.05.

## Discussion

The patients in this study cohort, more than one-quarter of whom were dependent on catecholamine infusion at the time of transfer, were younger and had higher severities of HF compared to the patients of other research studies on HF^14–16^. Georgiopoulou et al. analyzed a cohort of patients with HF referred for transplant evaluation who were slightly older and who had less severe HF compared to the patients in our study^17^. In our study, the patients from HBHs tended to have a poorer nutritional status and higher rate of catecholamine infusion during transfer than the patients from LBHs. In addition, an ICD or CRTD implantation was frequently performed before transfer for the patients from HBHs in our study. As a result, the patients from HBHs had a higher risk of experiencing the primary outcome, which was a composite of LVAD implantation and all-cause death. However, HBHs and the occurrence of the primary outcome did not show a statistically significant correlation in the multivariate analysis.

There are no established criteria for referral to specialized affiliations capable of performing LVAD or HTx. Therefore, the timing of referral to these specialized affiliations has been varied and wide-ranging. Indeed, a delay in referral can lead to a poorer prognosis in HF patients^9^. Therefore, investigating the appropriate timing for referral is imperative for improving the prognosis of advanced HF patients.

Approximately 10 centers in the Kanto area, which is 30,000 km^2^ and is located around Tokyo, perform LVAD interventions. The medical centers in this area are concentrated mainly around Tokyo compared with other sections, which suggests that the Kanto area is unevenly distributed compared with other areas in Japan. The patients transferred to our hospital usually came from hospitals in the Kanto area. This study included 186 patients with advanced HF from 116 hospitals, 50 of which have 500 beds or more and 66 of which have fewer than 500 beds.

We focused on the bed volume of hospitals in this study. However, hospitals also can be classified according to the hospital site (urban or rural), type of hospital facility, and funding background. The use of bed volume as a surrogate for hospital facility might be the simplest and easiest classification method, and we addressed the bed volume of hospitals classification in the analysis of the current study. There are several reports that demonstrated an association between hospital bed volume and clinical course of patients^10,11^, which might somewhat suggest the validity of this method. The cutoff value of 500 was calculated using the median of the referral hospital (504) in this study, and it corresponds to the criteria for large hospitals (500 beds) according to guidelines set by the American Hospital Association^18^. In contrast, another important measure that might reflect the scale of hospital is the “annual count of patients with a specific disease.” Indeed, several reports have demonstrated an association between HBH status and low mortality rate, and this trend is enhanced in patients who underwent major surgeries and cardiovascular procedures^19–21^. In addition, Kumbhani et al. demonstrated a weak association between HBH status and lower risk of short-term mortality in patients with HF^22^. However, our study analyzes referred patients, which do not have the same meaning as hospitalized patients in the referrer hospital, and there are few reports that have analyzed the association between referred patients and the referring hospital. We also compared the occurrence rates of the primary outcome and secondary outcome after classifying the hospitals into university hospitals and others or urban and rural hospitals (Supplementary Fig. 1, 2). However, these stratifications did not effectively classify patients with low and high risks.

According to the medication management for the patients, there were no significant differences in the medications taken for HF between the patients from the HBHs and patients from the LBHs, which is a result that is consistent with the results of previous studies^23^. In contrast, the finding that there was a large difference in the use of device therapies, such as CRTD and ICD, between the HBHs and LBHs suggests that hospitals with greater experience provide more advanced procedures.

The patient eligibility assessment for selecting patients with LVAD or HTx generally includes cardiopulmonary exercise testing, a comprehensive risk score, an evaluation of end-stage organ failure, and data on right heart catheterization^24^. Cardiopulmonary exercise testing can be performed only when patients are in a stable condition, so this evaluation was not useful for our cohort. In addition, there are several simple strategies for selecting suitable candidates for HTx or LVAD therapy without utilizing these data and measures. Thorvaldsen et al. demonstrated that risk factors such as low systolic blood pressure, renal dysfunction, anemia, and medication status can efficiently predict HTx/LVAD candidacy potential^25^. The study by Kagogeropoulos, which included patients with HF severities most similar to those in the current study, demonstrated that progression of HF to stage D is associated with a lower LVEF, lower blood pressure, and renal and hepatic dysfunction^26^. The BNP levels of patients in the present study likely reflect the effects of invasive and noninvasive treatments. This measure may be substantially affected by the timing of these treatments, which lessens the utility of this parameter^27^.

Some reports have noted that being referred to a HF center too late corresponds with a poor prognosis^9^. However, there was no significant difference in the survival rate between patients transferred from HBHs and those transferred from LBHs in this study. Indeed, the patients from HBHs had a longer disease history than did those from LBHs. Therefore, it is likely that the patients from HBHs endured a longer time without LVAD implantation than did those with from LBHs. Furthermore, differences in clinical characteristics between the patients from HBHs and LBHs were demonstrated for markers of nutrition and inflammation. A long duration of heart disease corresponded with a poorer nutritional state and higher degree of inflammation. We previously reported that a poor nutritional state with presence of inflammation corresponds to a poor clinical course following LVAD implantation^28,29^. Further recruitment of study patients and a more concise follow-up of LVAD complications would demonstrate an inferior survival and higher occurrence of LVAD-associated complications after LVAD implantation in patients from HBHs compared to patients from LBHs. However, this does not suggest that the timing of referral from HBHs was late. The factor of referral timing and HBH status should also be analyzed to elucidate appropriate patient allocation among various medical facilities. In addition, more patients should be evaluated to confirm the association found between survival after LVAD implantation and referral timing.

Many studies have analyzed prognostic factors in patients with HF^30,31^. However, there have been several differences among different subgroups of patients with HF, such as differences in their background characteristics or baseline cardiac function and etiology of HF. Therefore, the results of each study should be considered as subject to selection bias. In addition, the primary outcome of medical intractability, which includes LVAD implantation and death, was a determining factor. The present study has some similarities with the Kalogeropoulos study, which produced data on the progression of HF to stage D^26^, and with the Lanfear study, which examined LVAD-free survival^32^. Moreover, the treatment strategies of LVAD or HTx are limited to patients who meet the prescribed requirements of HTx candidacy, including age limitations, renal or liver function, and an absence of malignancy or active infection, which greatly affects the characteristics of the clinical course of disease.

This study has several limitations. The study population was comparatively small, and the study was performed at a single tertiary referral center in Japan, which might lessen the validity and importance of this study. In this cohort, the percentage of patients who were receiving beta-blockers or renin–angiotensin system antagonists was significantly lower than that of those in other studies^24^. However, this does not reflect the underutilization of appropriate medication for HF but rather indicates the difficulties of the administration of these medications due to patients having severe hypotension. Indeed, 32% of patients in the present study, which is significantly higher than that in previous studies, had a systolic blood pressure < 90 mmHg. In addition, the study design is limited in the evaluation of the effect of the medications. In this study, we analyzed only patients who were referred to our hospital. However, whether the referral was appropriate can be only sufficiently investigated after all patients, including both those referred and not referred, are analyzed. In addition, information is lacking in terms of the clinical course before patient transfer, such as transfer from LBHs to HBHs, which might also limit the validity of this study. The cultural context of LVAD and HTx in Japan also might affect the results of this study. For example, the implantation of LVAD is limited to functioning as a bridge to transplantation, and HTx is available only after a long duration of LVAD support. This substantially reduces the generalizability of the results of the present study. In addition, the high risk of complications, such as bleeding or thrombosis, from LVAD implantation makes its implementation and use more difficult. Therefore, there would be some possibility that factors other than those associated with advanced HF are considered in whether the patient receives LVAD implantation or HTx.

In conclusion, the patients referred to our center from HBHs had a higher risk of experiencing the primary outcome than did those referred to our center from LBHs. However, the association between HBH status and the outcomes was not significant in the multivariate analysis. In contrast, catecholamine support on transfer, long disease duration and low systolic pressure were independent predictors of the outcomes, and these should be considered as markers of advanced HF and justification for referral to an advanced HF center, irrespective of the bed volume of the referring hospital.

## Supplementary information

Supplementary Figures.
