# Supplementary material for: Differences in the prognoses of patients referred to an advanced heart failure center from hospitals with different bed volumes
Source: Sci Rep. 2020 Dec 3;10:21071. doi: 10.1038/s41598-020-78162-z (PMC7713124; doi:10.1038/s41598-020-78162-z)

**Different Characteristics and Prognoses of Referred Patients towards Advanced Heart Failure Center from Hospitals with Different Bed Volumes**

Koichi Narita, MD ^1^, Eisuke Amiya, MD, PhD ^1,2^, Masaru Hatano, MD, PhD ^1,2^, Junichi Ishida, MD, PhD ^1^, Hisataka Maki, MD, PhD ^5^, Shun Minatsuki, MD, PhD ^1^, Masaki Tsuji, MD, PhD ^1^, Akihito Saito, MD, PhD ^1^, Chie Bujo, MD, PhD ^1^, Satoshi Ishii, MD ^1^, Nobutaka Kakuda, MD ^1^, Mai Shimbo, MD, PhD ^1^, Yumiko Hosoya, MD, PhD ^1,2^, Miyoko Endo ^4^, Yukie Kagami ^4^, Hiroko Imai ^4^, Yoshifumi Itoda, MD, PhD ^3^, Masahiko Ando, MD, PhD ^3^, Shogo Shimada, MD, PhD ^3^, Osamu Kinoshita, MD, PhD ^3^, Minoru Ono, MD, PhD ^3^, Issei Komuro, MD, PhD ^1^

1. Department of Cardiovascular Medicine, Graduate School of Medicine, The University of Tokyo, Hongo 7-3-1, Bunkyo-ku, Tokyo 113-8655, Japan
2. Department of Therapeutic Strategy for Heart Failure, The University of Tokyo, Hongo 7-3-1, Bunkyo-ku, Tokyo 113-8655, Japan
3. Department of Cardiac Surgery, Graduate School of Medicine, The University of Tokyo, Hongo 7-3-1, Bunkyo-ku, Tokyo 113-8655, Japan
4. Department of Organ Transplantation, Graduate School of Medicine, The University of Tokyo, Hongo 7-3-1, Bunkyo-ku, Tokyo 113-8655, Japan
5. Department of Cardiovascular Medicine, Saitama Medical Center, Jichi Medical University, 1-847 Amanuma, Omiya-ku, Saitama City, 330-8503 Saitama, Japan

**Supplementary figure 1**: a) Difference in event free survival curve of primary event including left ventricular assist device implantation and death between patients from university hospitals and others. b) Difference in free survival curve between patients from university hospitals and others.


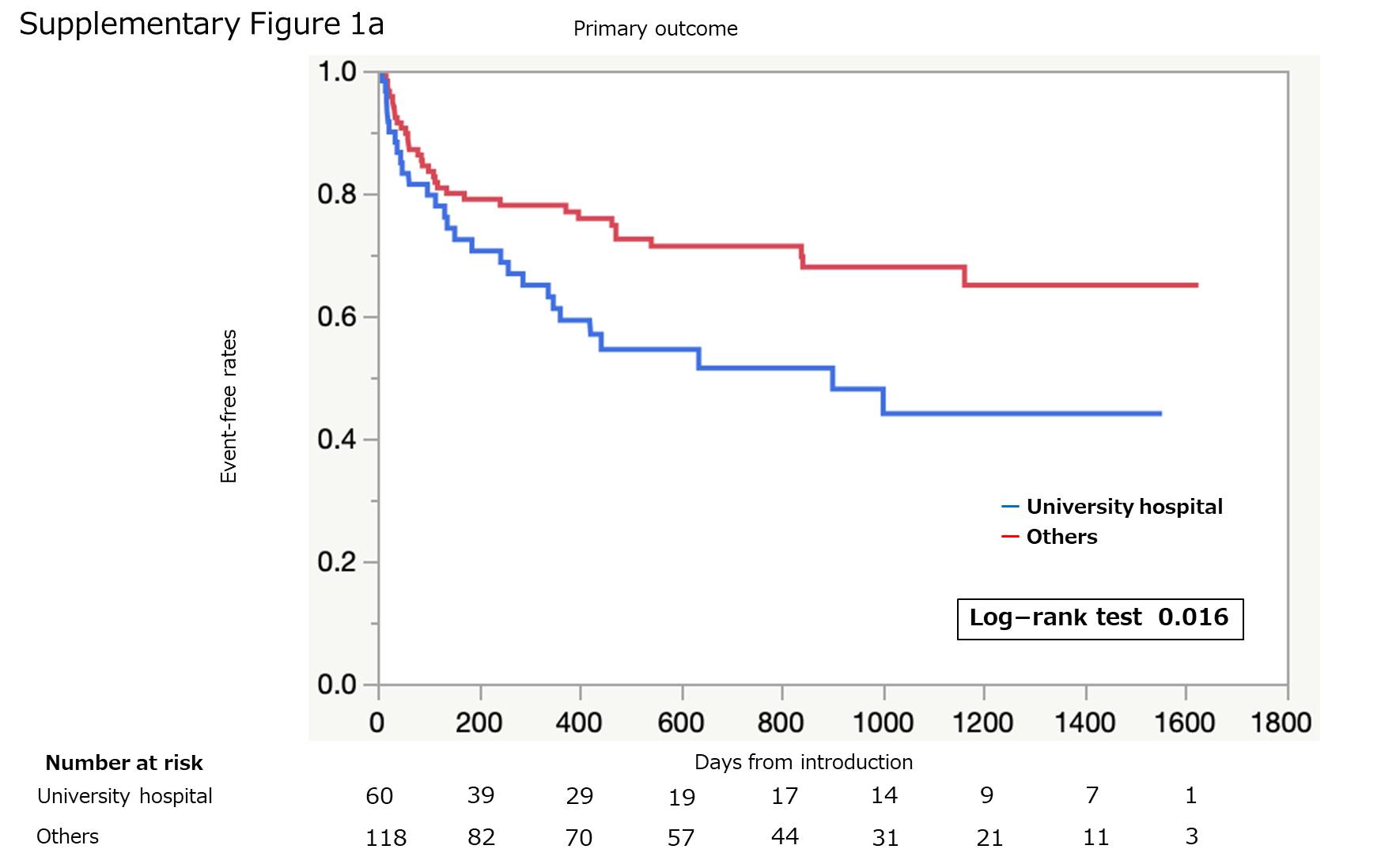


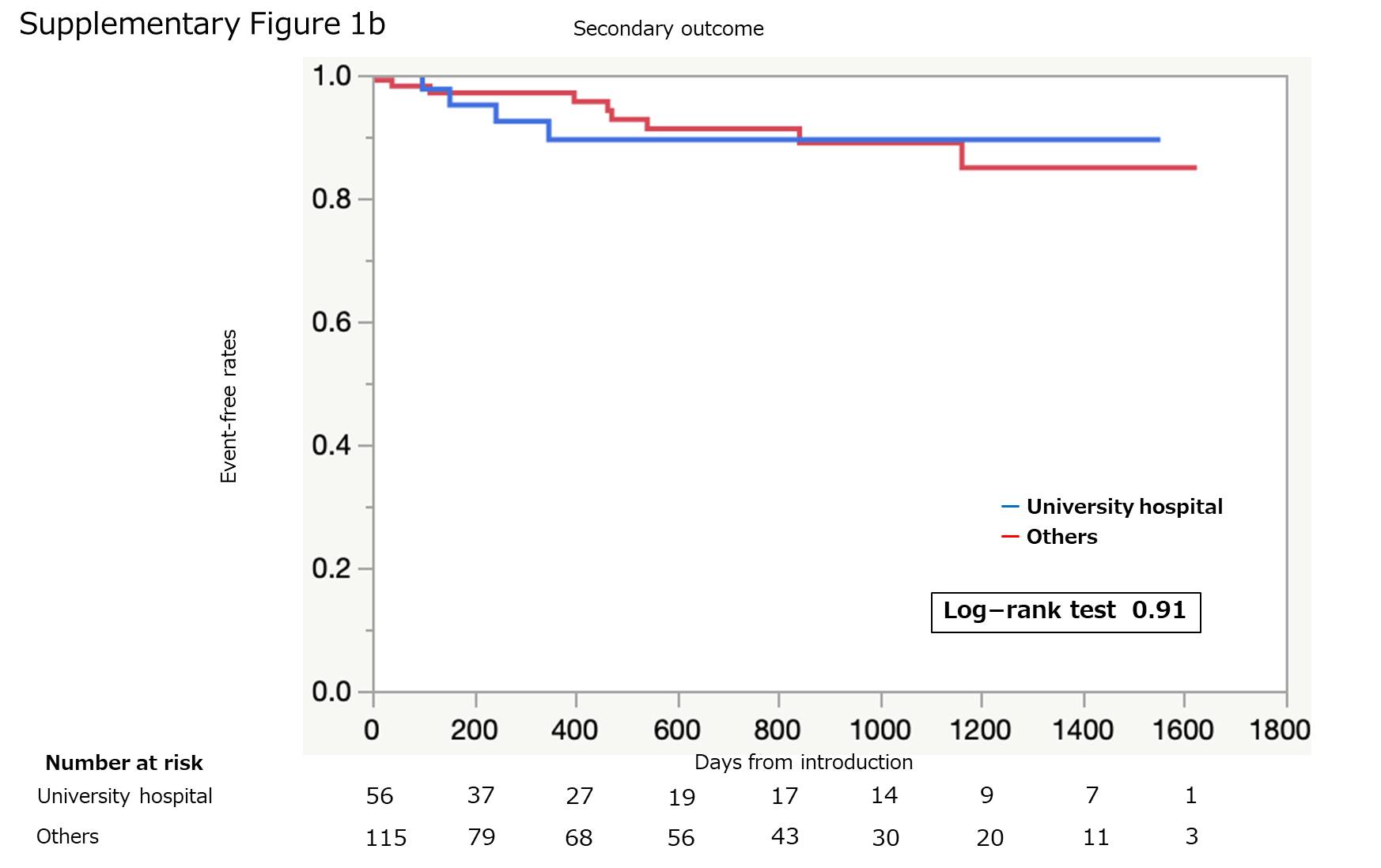


**Supplementary figure 2**: a) Difference in event free survival curve of primary event including left ventricular assist device implantation and death between patients from urban and rural hospitals. b) Difference in free survival curve between patients from urban and rural hospitals.


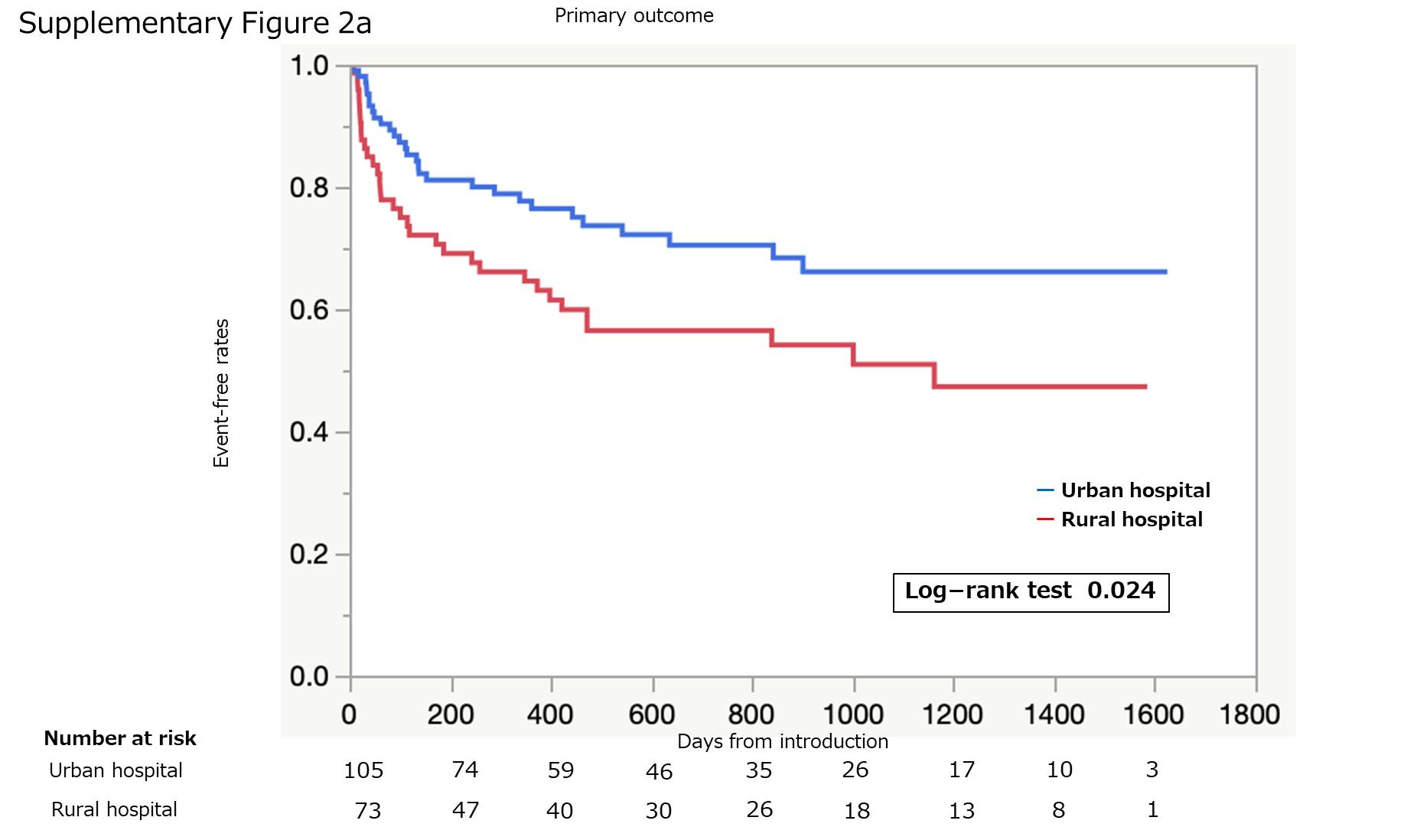


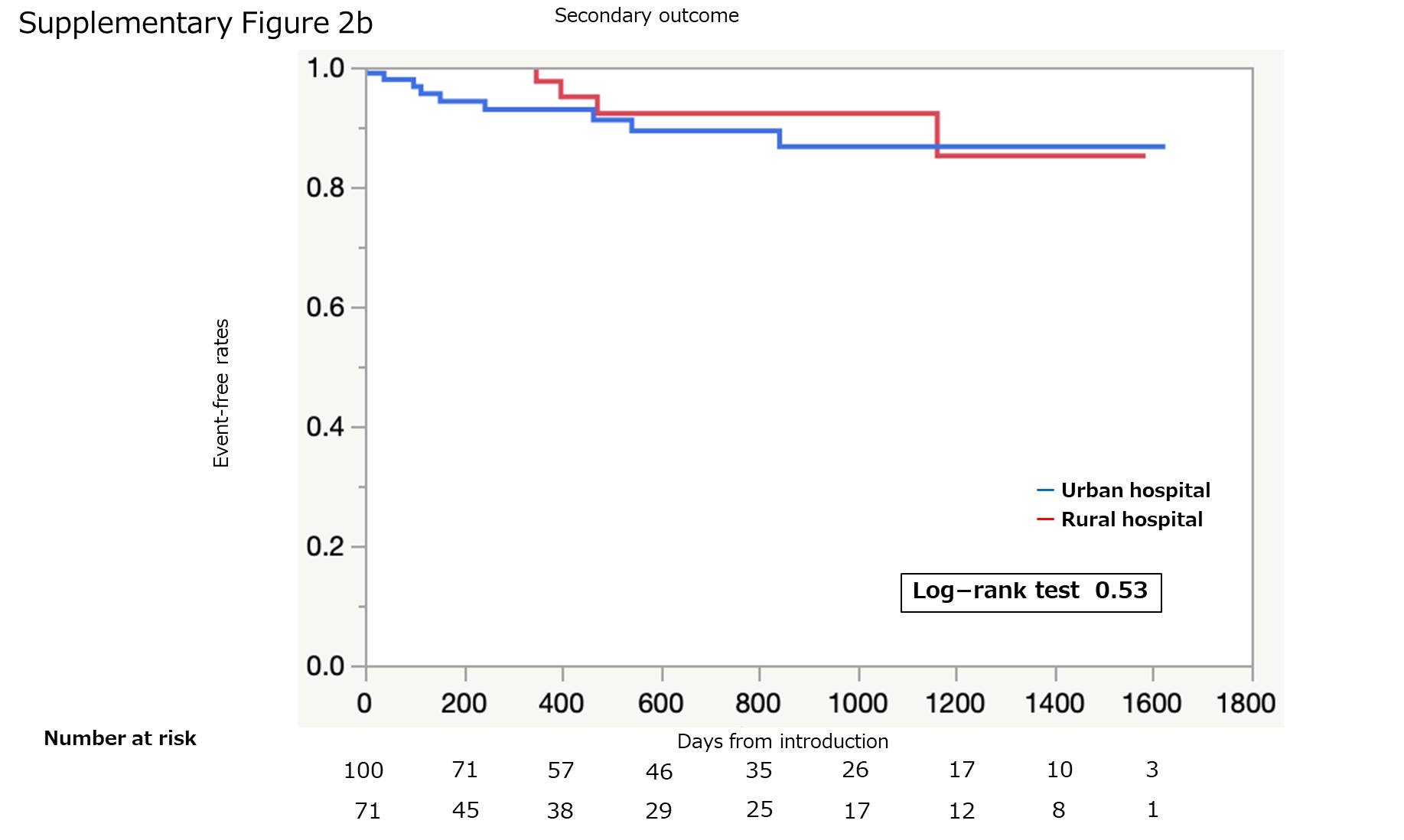

Supplement: Supplementary file 1 — Supplementary Figures. [file 41598_2020_78162_MOESM1_ESM.docx]
